# Effectiveness and Appropriateness of mHealth Interventions for Maternal and Child Health: Systematic Review

**DOI:** 10.2196/mhealth.8998

**Published:** 2018-01-09

**Authors:** Huan Chen, Yanling Chai, Le Dong, Wenyi Niu, Puhong Zhang

**Affiliations:** ^1^ The George Institute for Global Health Peking University Health Science Center Beijing China; ^2^ School of Public Health Peking University Health Science Center Beijing China; ^3^ School of Public Health Hebei Medical University Shijiazhuang China; ^4^ Faculty of Medicine University of New South Wales Sydney Australia

**Keywords:** telemedicine, maternal health, child health

## Abstract

**Background:**

The application of mobile health (mHealth) technology in reproductive, maternal, newborn, and child health (RMNCH) is increasing worldwide. However, best practice and the most effective mHealth interventions have not been reviewed systematically.

**Objective:**

A systematic review and meta-analysis of studies of mHealth interventions for RMNCH around the world were conducted to investigate their characteristics as well as the features and effectiveness of mHealth interventions.

**Methods:**

Studies of mHealth interventions for RMNCH between January 2011 and December 2016 were retrieved from 6 databases (PubMed, EMBASE, Global Health, China National Knowledge Infrastructure, VIP Database for Chinese Technical Periodicals, and Wanfang Data Knowledge Service Medium). Comparable studies were included in a random-effects meta-analysis for both exclusive breastfeeding (EBF) and antenatal checks (ANC). Descriptive analyses were conducted for mHealth studies with a range of study designs.

**Results:**

Analyses of 245 studies were included, including 51 randomized controlled trials (RCTs). Results showed that there are increasing numbers of studies on mHealth interventions for RMNCH. Although 2 meta-analysis, one with 2 RCTs on EBF (odds ratio [OR] 2.03, 95% CI 1.34-3.08, I^2^=25%) and the other with 3 RCTs on ANC (OR 1.43, 95% CI 1.13-1.79, I^2^=78%), showed that mHealth interventions are more effective than usual care, almost half (43%) of RCTs showed negative or unclear results on mHealth interventions. Functions described in mHealth interventions were diverse, and the health stages covered were broad. However, single function or single stage appeared to be dominant among mHealth interventions compared with multiple functions or stages.

**Conclusions:**

More rigorous evaluations are needed to draw consistent conclusions and to analyze mHealth products with multiple functions, especially those popular in the app markets.

## Introduction

Reproductive, maternal, newborn, and child health (RMNCH) has improved dramatically in the past two decades, according to a World Health Organization (WHO) report in 2015 [1]. However, there are new challenges, partly because of the changing burden of diseases (such as the increasing prevalence of noncommunicable diseases [NCDs]). Meanwhile, the needs of RMNCH, such as control of infectious disease and ensuring a safe pregnancy, continue to be relevant. Issues such as limited resources and engagement of patients in their health management remain challenges for the improvement of RMNCH health services worldwide [2-5].

In recent years, the rapid development of information and communication technologies (ICTs) in health care worldwide has led to the development of *mobile health (mHealth)* and enabled substantial change in the provision of health services [6,7]. mHealth technology is well suited to designing a patient-centered health service that increases the role of patients in medical treatment and encourages a degree of self-management, which is particularly important for a long-term chronic condition. Moreover, the widespread availability of ICT infrastructure in resource-limited settings provides access to high-quality health information and, in general, requires less staff and specialized health professionals [6,8]. A large number of RMNCH mobile applications (App), sensors, and wearable devices have been developed recently and are currently on the market, with diverse functions, ranging from bio-data monitoring to decision-making assistance [9-12].

Along with the boom of mHealth interventions in RMNCH, the number of studies to describe the development and evaluation of individual interventions is increasing. However, they are not yet extensive enough to provide adequate information to health professionals in making informed decisions about the best apps for particular health issues and situations. The existing systematic reviews either tend to focus on the effectiveness of mHealth interventions in the developing world, such as reviews by Lee et al [13], Sondaal et al [14], and Dahdah et al [15], or they focus on a single condition, such as a study on mHealth interventions for psychiatric conditions in children by Archangeli et al [16]. It seems that no study yet has described the features of RMNCH-related mHealth interventions comprehensively. Therefore, to meet this need, we conducted a systematic review and a meta-analysis of studies of RMNCH-related mHealth interventions around the world to investigate their appropriateness.

## Methods

### Search Strategies and Selection Criteria

We followed the methods detailed in a peer-reviewed systematic review protocol that is registered with International Prospective Register of Systematic Reviews (PROSPERO; CRD42017055570).

Three relevant English databases (PubMed, EMBASE, and Global Health) and 3 major Chinese databases (China National Knowledge Infrastructure, VIP Database for Chinese Technical Periodicals, and Wanfang Data Knowledge Service Medium) were searched. The search terms comprised key words from the following 3 dimensions: mHealth, maternal health care, and child health care. The searching strategy for each database was developed on the basis of key words identified from the literature and rules of subject headings in each database. Multimedia Appendix 1 shows details of the search terms used.

There has been significant development of mHealth [8] in medicine. This research paper examined studies in English and Chinese published between 1 January 2011 and 31 December 2016 (due to the language ability of the researchers). In addition, the references of included papers were reviewed to identify relevant papers.

Studies were eligible for inclusion if they aimed at improving RMNCH and studied interventions were conducted through mobile phone or tablet. The researchers focused on children under 6 years, as they are the most vulnerable group and a major target of mHealth interventions. Studies were excluded if their interventions were phone call alone, or functions and implementation were not clearly described, or they were descriptions of information technology. Systematic reviews or commentaries were also excluded, but kept as references. To achieve high sensitivity on search terms (but the accuracy was relatively low), no restriction was placed on study design and disease type. The corresponding authors were contacted if descriptions of interventions or studies were not clear enough for inclusion or exclusion.

All the searches were conducted on 21 February 2017. Searches were done independently by 2 reviewers, and a supervisor was invited for independent arbitration where consensus was not reached.

### Data Extraction

The following types of data were extracted: (1) basic information of research, such as the author, publication year, and the country and region where the interventions were delivered; (2) the target population, health care stages, and the health issue corresponding to a particular intervention; (3) the type of mHealth medium (App or short message service [SMS]), and the description and function of the health intervention; (4) the study design and the number of participants given the health interventions; and (5) the primary outcome and the results of the study. The data extraction and quality assessment were processed by 2 independent reviewers, and any disagreement was resolved by a supervisor. The categorization of functions was based on mHealth and ICT Framework for mHealth innovations in the RMNCH field, which had 12 common mHealth applications used as health system strengthening innovations [11].

### Data Analysis

The studies were not limited to randomized controlled trials (RCTs), as the researchers aimed to analyze and present the characteristics of RMNCH-related mHealth interventions described in all the reviewed studies. The descriptive analysis of the main characteristics and the key findings were processed and presented. For RCTs, we presented their results on effectiveness as categorical variables. Studies that showed significantly better effectiveness in the intervention group compared with the control group were recorded as positive or recorded as negative or not clear if no statistical data were presented.

Substantial heterogeneity existed among the studies. As a result, the researchers were only able to perform random-effects meta-analysis using the inverse variance method for the 5 comparable studies, 2 studies on exclusive breastfeeding (EBF) and 3 studies on antenatal check (ANC). Meta-analyses were undertaken and the bias of the 5 studies was evaluated using Review Manager 5.3 (Cochrane Collaboration).

## Results

**Figure 1:**
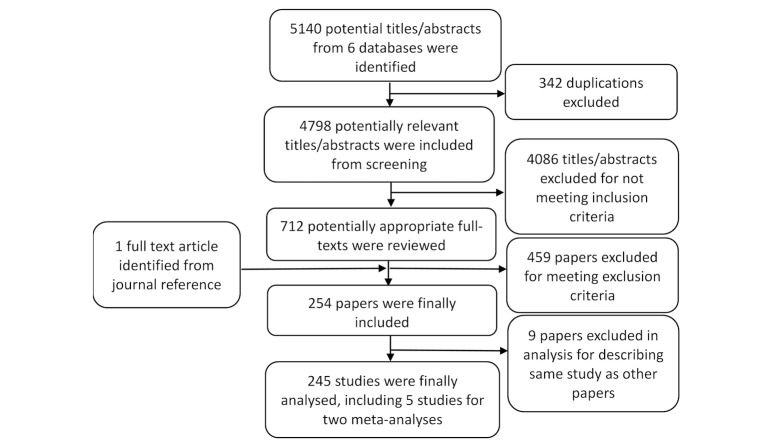
Identification process for eligible studies.

From 6 databases, 5140 papers were identified in the initial search for screening. Finally, 245 published papers (studies) were included in this systematic review, among which 20.8% (51/245) studies were RCTs and 24.9% (61/245) were quasi-experimental studies. Two studies [17,18] on EBF and three studies [19-21] on ANC were included in the meta-analysis. The search process is shown in Figure 1.

**Figure 2:**
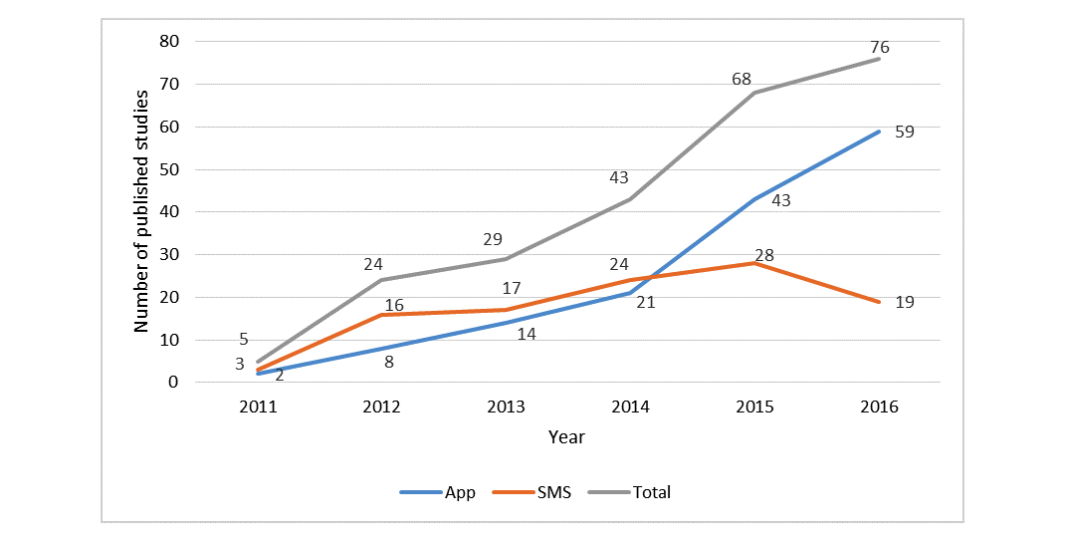
Trend of studies published from 2011 to 2016. Interventions combined with SMS and App were counted twice in each medium.

Figure 2 shows that the number of publications on mHealth in RMNCH increased significantly from 2011 to 2016. Among the 245 studies, interventions in 40% (98/245) studies were carried out through the SMS medium, 56.3% (138/245) through mobile or tablet-based apps (including 2 Web-based Apps), and 3.8% (9/245) combining SMS and App concurrently. The number of studies on the App also saw a steady increase from 2011 to 2016, whereas the number of studies on SMS increased slightly from 2011 to 2015 and then decreased from 2015 to 2016.

The findings and characteristics of the studies are presented in Table 1, including geographic distribution, medium, target population, health care stage, health issue addressed, and study design. The 245 studies came from 46 countries and 6 regions, and studies from the West Pacific, the Americas, and Africa regions accounted for 78.3% of all studies.

The targeted populations of mHealth interventions included both health service beneficiaries and providers. However, the number of studies targeting health service beneficiaries (n=176) was 3 times higher than that of health service providers (n=55). Only a few (n=14) considered both beneficiaries and providers.

This study divided RMNCH care into 5 stages, namely, prepregnancy, pregnancy, delivery, postpartum, and childcare. Although 76.3% (187/245) of the studies investigated mHealth interventions for childcare and pregnancy, only 6.5% (16/245) studies paid attention to delivery and postpartum stages. Ninety-one percent of mHealth interventions (224/245) focused on single stage, whereas 8.5% (21/245) of interventions designed for 2 and/or more stages at the same time.

The functions of mHealth interventions were categorized into 16 types (Table 2), which largely overlap with the ICT Framework, except the functions that optimize hospital service flow, such as setting an appointment (to set doctor’s appointments with hospital) and laboratory results (to check laboratory results through an App linked with the hospitals’ information system). Meanwhile, functions relating to human resource management and supply chain management described by the ICT framework were not identified in this study. Health education or promotion (to provide users with health information and lifestyle advice), physical or bio data monitoring (to monitor physical or bio data of patients in distance and to adjust for treatment in real time, especially for chronic conditions), and reminders (to remind users for antenatal checks, the ovulation time, medication, etc) are the most commonly used functions. Apart from health education or promotion, there is a difference in the most frequently adopted functions among App and SMS interventions. That is, the reminders (27.1%) and data collection and management (to collect data for research or administrative purposes) (15.9%) ranked second and third most popular functions in SMS interventions, respectively, whereas physical or bio data monitoring (36.1%) and counseling (to consult a health professional directly) (17.7%) ranked second and third most popular in App interventions, respectively. mHealth interventions with complex algorithms were more frequently observed in Apps compared with SMSs, such as decision support and diagnosis. The number of mHealth interventions with single functions was more than twice of that with multi-functions (≥2 functions), which were more frequently seen in the App.

Studies on SMS and/or App interventions were distributed unevenly among regions. In Africa, the number of SMS-based studies (65.5%) was about 3 times higher than that of App-based studies (32.7%), whereas App-based studies played a dominant role in South-East Asia (80.0%), Europe (75.0%), the Eastern Mediterranean (66.7%), and the Americas (59.7%). Meanwhile, the most frequently adopted functions in each region were associated with the type of mediums (SMS and/or App) dominantly applied in that region.

For the 51 identified RCTs, we presented their results in Table 3 with respect to whether the results showed that mHealth interventions were significantly effective. More than half (n=29) of the studies had positive results supporting the effectiveness of mHealth interventions and 43.1% (n=22) had negative or unclear results. Details about the effectiveness of mHealth interventions based on different mediums, functions, health issues, and the stages are shown in Table 3.

**Figure 3:**
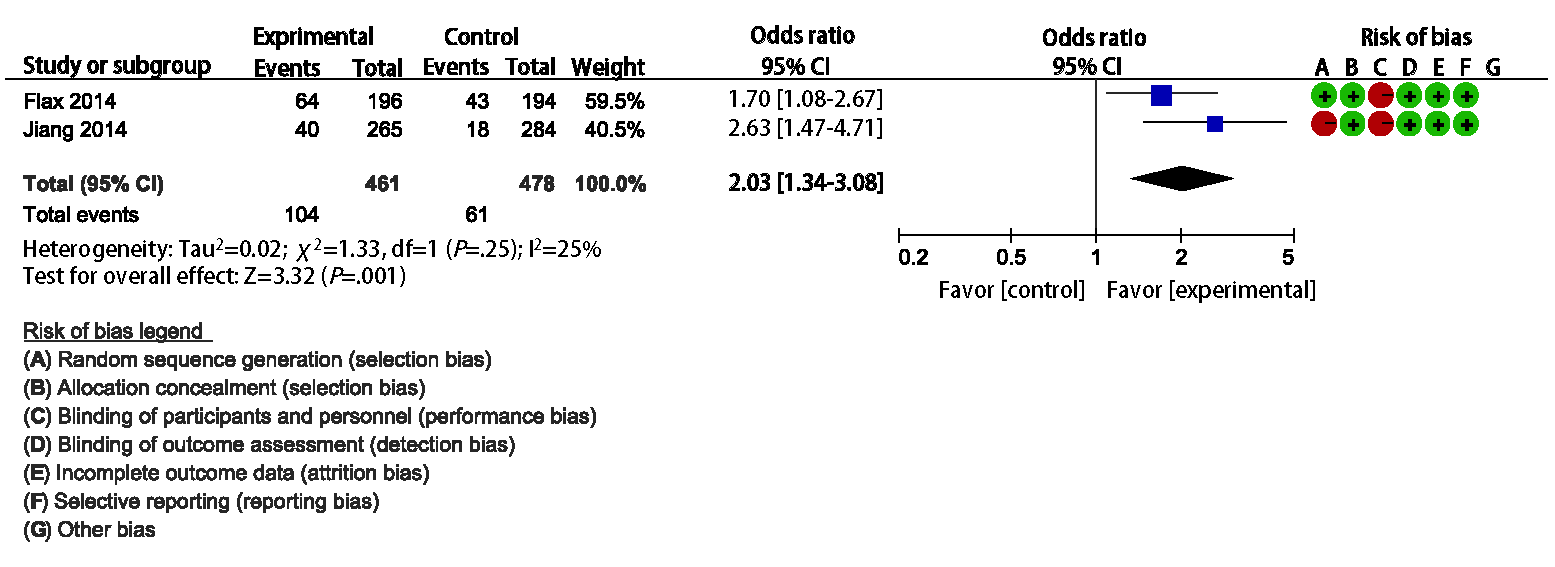
Meta-analysis of the effect of mHealth intervention versus routine prenatal care on exclusive breastfeeding for 6 months based on two studies undertaken in Nigeria and China.

Two studies (Flax et al [17] and Jiang et al [18]) compared the effect of mHealth interventions using SMS compared with routine health care, encouraging breastfeeding in Nigeria and China. The results of both trials showed that the rates of EBF for 6 months were higher in the intervention group than in the control group. We undertook meta-analysis of the effect of mHealth intervention versus routine health care on EBF for 6 months. The merged estimates showed that the rate of EBF for 6 months was higher in the mHealth intervention groups compared with the control group (OR 2.03, 95% CI 1.34-3.08, I^2^=25%; Figure 3).

**Figure 4:**
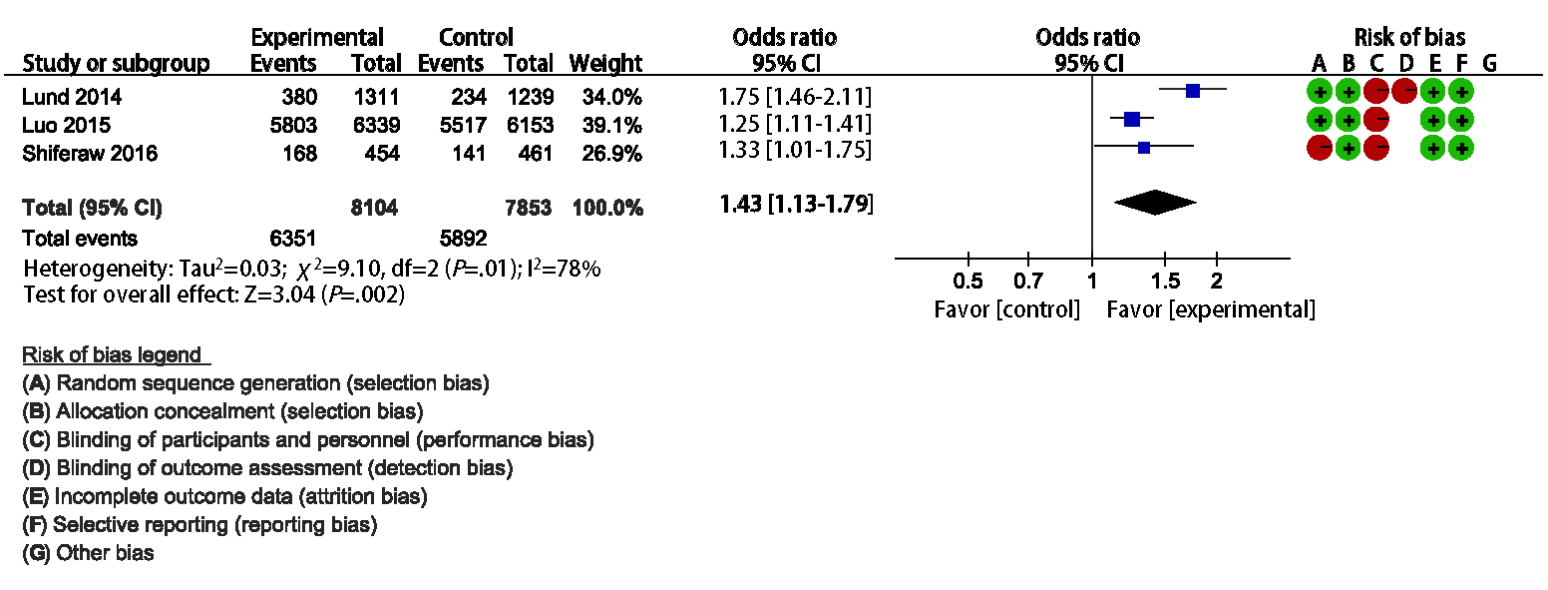
Meta-analysis of the effect of mHealth intervention versus routine prenatal care on four or more antenatal check rates based on three studies undertaken in Zanzibar, China, and Ethiopia.

Lund et al [19], Luo et al [20], and Shiferaw et al [21] compared the effect of mHealth interventions versus routine care on ANC in Zanzibar (SMS), China (SMS), and Ethiopia (App), respectively. The results of all trials showed that the rates of 4 or more ANCs were higher in the mHealth group than in the control group. The merged estimates from the meta-analysis showed that the rates of 4 or more ANCs (OR 1.43, 95% CI 1.13-1.79, I^2^=78%; Figure 4) were higher in the mHealth intervention groups than in the control groups.

**Table 1:** Characteristics of included studies.

Category			Studies, n (%)
**Region**			
	The Americas		67 (27.3)
	Europe		32 (13.1)
	The Western Pacific		70 (28.6)
	South-East Asia		15 (6.1)
	The Eastern Mediterranean		6 (2.4)
	Africa		55 (22.4)
**Health issues^a^**			
	Infectious diseases		28 (11.4)
	Chronic diseases		43 (17.6)
	Mental and behavioral disorders		11 (4.5)
	Essential RMNCH^b^ issues		16 (66.5)
**Study design^c^**			
	mHealth product description		87 (35.5)
	Quasi-experiment		61 (24.9)
	RCT^d^		51 (20.8)
	Cross-sectional study		21 (8.6)
	RCT protocol		19 (7.7)
	Qualitative study		5 (2.0)
	Case report		1 (0.4)
**Medium**			
	SMS^e^		98 (40.0)
	App		138 (56.3)
	SMS and App		9 (3.8)
**Target population**			
	**Health service beneficiaries**		176 (71.8)
		Women^f^	94 (38.4)
		Parents	76 (31.0)
		Children	11 (4.5)
	**Health service providers**		55 (22.4)
		Health professionals	29 (11.8)
		Health workers and volunteers	24 (9.8)
		Administrators	3 (1.2)
	Beneficiaries and providers		14 (5.7)
**Health care stages**			
	Prepregnancy		21 (8.6)
	Pregnancy		68 (27.8)
	Delivery and postpartum		16 (6.5)
	Childcare		119 (48.6)
	Multi-stages		21 (8.6)

^a^Forty-seven types of health issues were identified from included studies and divided into 4 categories based on the 10^th^version of the International Statistical Classification of Diseases and Related Health Problems (International Classification of Diseases-10) and data availability. The details on these health issues can be found in Multimedia Appendix 2.

^b^RMNCH: Reproductive, maternal, newborn, and child health.

^c^Details on study design can be found in Multimedia Appendix 3.

^d^RCT: randomized controlled trial.

^e^SMS: short message service.

^f^Women including women at child-bearing age, pregnant women, and perinatal women.

**Table 2:** Functions of mHealth interventions delivered by short message service and App.

Function^a^	Total, N (%)	SMS^b^, n (%)	App, n (%)	
Single function	162 (66.1)	75 (46.3)	92 (56.8)	
Two functions	69 (28.2)	30 (43.5)	42 (60.9)	
Three and more functions	14 (5.7)	2 (14.3)	13 (92.9)	
Health education or promotion	110 (44.9)	60 (56.1)	53 (36.1)	
Physical or bio data monitoring	58 (23.7)	10 (9.3)	53 (36.1)	
Reminders	40 (16.3)	29 (27.1)	12 (8.2)	
Counseling	38 (15.5)	15 (14.0)	26 (17.7)	
Data collection and management	37 (15.1)	17 (15.9)	20 (13.6)	
Decision support and guideline	18 (7.3)	0 (0.0)	18 (12.2)	
Diagnosis and treatment	17 (6.9)	2 (1.9)	17 (11.6)	
Appointment making	11 (4.5)	4 (3.7)	7 (4.8)	
On-the-job training for health professionals	9 (3.7)	2 (1.9)	7 (4.8)	
Laboratory results	5 (2.0)	0 (0.0)	5 (3.4)	
Communication	4 (1.6)	1 (0.9)	3 (2.0)	
Payment	3 (1.2)	0 (0.0)	3 (2.0)	
Supervision and technical support	2 (0.8)	0 (0.0)	2 (1.4)	
Hospital guidelines	2 (0.8)	0 (0.0)	2 (1.4)	
Cash transfer	1 (0.4)	1 (0.9)	0 (0.0)	
Electronic health record check	1 (0.4)	0 (0.0)	1 (0.7)	

^a^Studies with multiple functions were counted repeatedly in each function category.

^b^SMS: short message service.

**Table 3:** mHealth interventions and results from randomized clinical trials.

Category		Total studies, N	RCTs^a^, n	RCTs with positive results, n (%)
Total		245	51	29 (56.9)
**Medium**				
	App	147	13	9 (69.2)
	SMS^b^	107	40	22 (55.0)
**Function**				
	Studies with multiple functions^c^	83	13	9 (69.2)
	Studies with single functions	162	38	20 (52.6)
**Health issue**				
	Essential RMNCH^d^ issues	163	37	21 (56.8)
	Other RMNCH-related diseases^e^	82	14	8 (57.1)
**Stages**				
	Prepregnancy	26	10	6 (60.0)
	Pregnancy	88	14	11 (78.6)
	Delivery and postpartum	28	4	3 (75.0)
	Child care	139	24	10 (41.7)

^a^RCTs: randomized controlled trials.

^b^SMS: short message service.

^c^Multiple functions refer to 2 or more functions, such as health education or promotion, physical or biodata monitoring, and reminders concurrently.

^d^RMNCH: reproductive, maternal, newborn, and child health.

^e^Other RMNCH-related diseases include infectious diseases, chronic diseases, and mental and behavioral disorders.

## Discussion

### Principal Findings

The study found a rapid increase in the number of publications on mHealth interventions for RMNCH, especially for those using App. However, the overall number of publications included (n=245) remained relatively small, among which only 51 RCTs were identified. Although 2 meta-analyses based on 2 studies on EBF and 3 studies on ANC showed positive evidences to support the effectiveness of mHealth interventions, almost half (43.2%) of RCTs showed negative or unclear results of mHealth interventions. In addition, the studies were too heterogeneous and dispersed to generate merged results for individual mHealth interventions on specific health issues. Among all interventions identified in this study, the ones designed for health care beneficiaries exceeded 3 times those for health care providers. mHealth interventions with a single function or stage were dominant among all identified studies.

mHealth interventions are popular worldwide with fast upgrades of technologies and improvement of communication infrastructure. However, the overall number of relevant publications remains relatively small compared with other interventions for RMNCH [22]. Consistent with Sahu’s finding [23], Apps seem to dominate the mHealth market compared with SMS, as they can provide multiple and complex functions simultaneously.

More than one-fifth of the studies (22.4%) were from Africa, which is comparable to that from Western Pacific (28.6%) and the Americas (27.3%). This can be partially explained by the rapid development of communication infrastructure and high ownership rate of mobile phones in Africa in recent years [24,25]. Tailored messages and other mHealth interventions through mobile phone can reach target populations and the hard-to-reach population more efficiently compared with traditional approaches.

Evidences on the effectiveness of single or multiple mHealth functions on individual or multiple health conditions were far from adequate. A high proportion of descriptive and observational studies and small number of high-quality RCTs identified indicate that evaluation of mHealth innovation designed for RMNCH is in its infancy. This led to a result that diversified outcome indicators and health issues were reported from a very limited number of RCTs, which placed huge challenges in acquiring merged effects of mHealth interventions.

Although the results of 2 meta-analyses on EBF [17,18] and ANC [19-21] supported the use of mHealth interventions, almost half of the RCTs still showed negative or inconclusive results of mHealth interventions, which could be explained in 3 ways. First, most RCTs with negative or inconclusive results have a small sample size (less than 200 people) or a short follow-up time (less than 6 months). Second, major outcome measurements, such as blood glucose and weight, are hard to be improved in a short follow-up period. Third, most RCTs were less rigorous in study design, as the evaluation of mHealth interventions is still at an early stage. Therefore, the results of these included studies need to be further tested to reduce the risk of bias derived from any inherent weakness in the study designs.

Although the number of high-quality RCTs identified for the health service provider–related functions was scarce, many quasi-experimental studies [26-29] provided some preliminary evidence in favor of functions, including decision support (to provide clinical guidelines for health professionals when necessary) and on-the-job training (to conduct trainings for health professionals to improve service quality). This may shed some light for future study, as providing technical support to health service provider is crucial in improving health care quality and retention of service providers from health system strengthening perspective.

Interventions delivered through SMS could only enable functions with simple algorithms, such as health education and reminders. One of our previous studies found that almost all existing RMNCH Apps in the Chinese market were embedded with multiple functions and covered multiple stages through a person’s life course. However, most of the studies included in this review focused on single function and stage, which reveals a gap between research and practice.

Apart from commonly concerned ANC and general childcare (such as feeding and immunization), studies from developing countries largely focused on infectious disease and essential maternal and childcare (such as human immunodeficiency virus [HIV] and diarrhea), whereas studies from developed countries explored more on noncommunicable diseases (such as asthma, gestational diabetes mellitus, and cancer). However, disease prevalence is changing and burden of NCDs is becoming devastating in those developing countries, according to WHO’s latest report on global disease burden [30,31]. Therefore, NCDs should be given more consideration when designing mHealth interventions and studies in developing countries.

This study also found that SMS was used more frequently in low-middle income countries compared with Apps, providing basic functions such as health education or promotion [32,33], reminder [34-36], and data collection [37,38]. RCTs on SMS showed some positive results in the prevention of mother-to-child transmission (PMTCT), childhood disease management, and family planning. However, the evidence is not adequate to draw conclusions that one function is more effective for a particular health condition when delivered through either SMS or App. When it comes to the selection of SMS over App, cost-effectiveness analysis need to be considered, as the cost for SMS-based functions usually is much cheaper than that for App-based functions [39].

### Conclusions

The major limitation of this study is that only 6 databases were searched. This can result in missing of high-quality RCTs on mHealth intervention for RMNCH, which may contribute to merged effect and lend more weight to the effectiveness of some mHealth interventions. In summary, published studies on RMNCH-related mHealth interventions are increasing, but have been far from adequate in evaluating the effectiveness of such interventions on individual health issues. More rigorous evaluations are needed to draw consistent conclusions. The studied mHealth interventions were relatively simple. More research is needed to evaluate mHealth products with multiple functions or stages, especially those popular outside clinical practice.

## Appendix

Multimedia Appendix 1 Search strategies.

Multimedia Appendix 2 Details of health issues targeted by mHealth interventions.

Multimedia Appendix 3 Study designs.

## Abbreviations

ANC: antenatal checks
EBF: exclusive breastfeeding
HIV: human immunodeficiency virus
ICT: information and communication technology
mHealth: mobile health
NCDs: noncommunicable diseases
OR: odds ratio
PMTCT: revention of mother-to-child transmission
RCT: randomized controlled trial
RMNCH: reproductive, maternal, newborn, and child health
SMS: short message service
WHO: World Health Organization
