# Supplementary material for: Effectiveness and Appropriateness of mHealth Interventions for Maternal and Child Health: Systematic Review
Source: JMIR Mhealth Uhealth. 2018 Jan 9;6(1):e7. doi: 10.2196/mhealth.8998 (PMC5780618; doi:10.2196/mhealth.8998)
Supplement: Multimedia Appendix 1 [file mhealth_v6i1e7_app1.pdf]

Appendix 1. Search strategies

**PubMed**		
	((((((((Mhealth) OR mobile health) OR ehealth) OR App) OR smartphone) OR iOS) OR android)) AND ((((((maternal health) OR maternal care) OR newborn) OR infant) OR child)) AND ("last 5 years"[PDat])	2207

**EMBASE**		
1	(mhealth or mobile health or App or smartphone or cellphone or ios or android).mp. [mp=title, abstract, heading word, drug trade name, original title, device manufacturer, drug manufacturer, device trade name, keyword](38858)	38858
2	exp mobile Application/ or exp mobile phone/(12333)	12333
3	1 or 2 (48391)	48391
4	(maternal health or maternal care or pregnan* or antenatal or postpartum or delivery).mp. [mp=title, abstract, heading word, drug trade name, original title, device manufacturer, drug manufacturer, device trade name, keyword] (1369513)	1369513
5	exp maternal care/ (34324)	34324
6	4 or 5 (1375749)	1375749
7	((newborn* or infant* or child* or baby*) adj3 (health or care or development)).mp. [mp=title, abstract, heading word, drug trade name, original title, device manufacturer, drug manufacturer, device trade name, keyword] (231260)	231260
8	exp child care/ (53041)	53041
9	7 or 8 (247093)	247093
10	exp family planning/ (34236)	34236
11	6 or 9 or 10 (1593372)	1593372
12	3 and 11 (3204)	3204
13	limit 12 to (human and english language and yr="2011 - 2016") (1939)	1939

**Global Health**		
1	(mhealth or mobile health or App or smartphone or cellphone or ios or android). mp. [mp=abstract, title, original title, broad terms, heading words, identifiers, cabicodes]	1624
2	exp mobile telephones/	972
3	1 or 2	2305
4	(maternal health or maternal care or pregnan* or perinatal or antenatal or postpartum or delivery).mp. [mp=abstract, title, original title, broad terms, heading words, identifiers, cabicodes]	136794
5	exp maternity/ or exp maternity services/	3671
6	exp pregnancy/ or exp sexual reproduction/ or exp birth/ or exp conception/ or exp gestation period/ or maternal nutrition/ or exp pregnancy complications/ or exp pregnancy diagnosis/ or exp prenatal care/ or exp reproductive health/	103310
7	family planning/ or exp contraception/ or exp reproductive health/	11202
8	((newborn* neonat* or infant* or child* or baby*) adj3 (health or care or growth or development)).mp. [mp=abstract, title, original title, broad terms, heading words, identifiers, cabicodes]	41228
9	exp childbirth/ or exp childhood diseases/ or children/ or exp preschool children/ or exp child care/ or exp child development/ or exp child feeding/or exp child health/ or child nutrition/ or exp infants/ or exp paediatrics/	348586
10	4 or 5 or 6 or 7 or 8 or 9	467875
11	3 and 10	569
12	limit 11 to (english language and yr="2011 - 2016")	457

**China National Knowledge Infrastructure**		
	(SU='mobile' + 'App' + 'cellphone') * (SU='maternal and child' + 'mother and baby' + 'pregnant and lying-in women' + 'pregnant women' + 'postpartum womem' + 'newborn' + 'infant') * (FT='health' + 'medical' + 'hospital' + 'health care' + 'grow up')	377

**VIP Database for Chinese Technical Periodicals**		
	(K=mobile +K=App +R=cellphone)*(K=maternal and child +K=mother and baby +K=pregnant and lying-in women +K=newborn +K=infant) *(FT= 'health' + 'medical' + 'hospital' + 'health care')	49

**Wanfang Data Knowledge Service Medium**		
	Title or keywords: ('mobile or App or cellphone') * Title or keywords:('maternal and	111

	child or mother and baby or pregnant and lying-in women or newborn' or 'infant') *<br>Title or keywords: ('health' or 'medical' or 'hospital' or 'health care' or 'grow up') *<br>Date:2011-2016
