# Supplementary material for: Effectiveness and Appropriateness of mHealth Interventions for Maternal and Child Health: Systematic Review
Source: JMIR Mhealth Uhealth. 2018 Jan 9;6(1):e7. doi: 10.2196/mhealth.8998 (PMC5780618; doi:10.2196/mhealth.8998)
Supplement: Multimedia Appendix 2 [file mhealth_v6i1e7_app2.pdf]

Appendix 2. Details of health issues targeted by mHealth interventions

Health issue	No. of Studies	Percentage (%)
**Infectious diseases**	**28**	**11.4**
Childhood disease management (multiple diseases)	13	5.3
PMTCT	8	3.3
HIV	5	2.0
Chagas disease	1	0.4
diarrhoea	1	0.4
**Chronic diseases**	**43**	**17.6**
Obesity	17	6.9
Respiratory diseases	3	1.2
Asthma	3	1.2
Oral health	3	1.2
Cancer	2	0.8
Hepatopathy	2	0.8
Amino acid metabolic disease	1	0.4
Malnutrition	1	0.4
Diabetes Mellitus	1	0.4
Neural development	1	0.4
Epilepsy	1	0.4
Chronic otitis media	1	0.4
Hearing disorders	1	0.4
Venous thromboembolism	1	0.4
Tonsillectomy	1	0.4
Nephropathy	1	0.4
Congenital heart disease	1	0.4
Dyslexic	1	0.4
Speech disorders	1	0.4
**Mental and behavioural disorders**	**11**	**4.5**
Mental disorders	7	2.9
Depression	3	1.2
Sleep	1	0.4
**Essential RMNCH issues**	**163**	**66.5**
Antenatal care	38	15.5
Immunization	24	9.8
Feeding and diet	20	8.2
General RMNCH care	19	7.8
Family planning	18	7.3
Gestational diabetes mellitus	14	5.7
Safety	5	2.0
Physical activity	4	1.6
Delivery	3	1.2
Neonatal care	3	1.2
General child care	3	1.2
Preeclampsia	2	0.8
Child growth	2	0.8

Medication usage	2	0.8
Parenting	2	0.8
Abortion	1	0.4
Postnatal care	1	0.4
Postnatal care and child care	1	0.4
Neonatal disease screening	1	0.4
**Total**	**245**	**100.0**
