# Supplementary material for: Effectiveness and Appropriateness of mHealth Interventions for Maternal and Child Health: Systematic Review
Source: JMIR Mhealth Uhealth. 2018 Jan 9;6(1):e7. doi: 10.2196/mhealth.8998 (PMC5780618; doi:10.2196/mhealth.8998)
Supplement: Multimedia Appendix 3 [file mhealth_v6i1e7_app3.pdf]

Appendix 3. Study designs

	Category	**Total**		**RCT**		**Quasi-experiment**		**RCT protocol**		**MHealth product description**		**Cross-sectional study**		**Qualitative study**		**Case report**	
		**N**	**%**	**N**	**%**	**N**	**%**	**N**	**%**	**N**	**%**	**N**	**%**	**N**	**%**	**N**	**%**
**Region**																	
	The Americas	67	27.3	19	28.4	15	22.4	0	0.0	26	38.8	5	7.5	1	1.5	1	1.5
	Europe	32	13.1	4	12.5	7	21.9	4	12.5	14	43.8	3	9.4	0	0.0	0	0.0
	The Western Pacific	70	28.6	14	20.0	15	21.4	10	14.3	27	38.6	3	4.3	1	1.4	0	0.0
	South-East Asia	15	6.1	0	0.0	7	46.7	0	0.0	6	40.0	2	13.3	0	0.0	0	0.0
	The Eastern Mediterranean	6	2.4	1	16.7	0	0.0	1	16.7	3	50.0	1	16.7	0	0.0	0	0.0
	Africa	55	22.4	13	23.6	17	30.9	4	7.3	11	20.0	7	12.7	3	5.5	0	0.0
**Medium**																	
	SMS	107	43.7	40	37.4	37	34.6	7	6.5	11	10.3	10	9.3	2	1.9	0	0.0
	App	147	60.0	13	8.8	27	18.4	12	8.2	79	53.7	12	8.2	3	2.0	1	0.7
**Function**																	
	**Function for health service beneficiaries**																
	Health education/promotion	110	44.9	32	29.1	28	25.5	9	8.2	34	30.9	4	3.6	3	2.7	0	0.0
	Physical data monitoring	58	23.7	6	10.3	8	13.8	3	5.2	35	60.3	6	10.3	0	0.0	0	0.0
	Reminders	40	16.3	14	35.0	10	25.0	7	17.5	7	17.5	2	5.0	0	0.0	0	0.0
	Diagnosis and treatment	17	6.9	2	11.8	4	23.5	3	17.6	7	41.2	0	0.0	0	0.0	1	5.9
	Appointment making	11	4.5	3	27.3	2	18.2	0	0.0	5	45.5	1	9.1	0	0.0	0	0.0
	Laboratory results	5	2.0	0	0.0	0	0.0	0	0.0	5	100.0	0	0.0	0	0.0	0	0.0
	Communication	4	1.6	0	0.0	1	25.0	1	25.0	2	50.0	0	0.0	0	0.0	0	0.0
	Payment	3	1.2	0	0.0	0	0.0	0	0.0	3	100.0	0	0.0	0	0.0	0	0.0
	Hospital guidelines	2	0.8	0	0.0	0	0.0	0	0.0	2	100.0	0	0.0	0	0.0	0	0.0
	Cash transfer	1	0.4	0	0.0	1	100.0	0	0.0	0	0.0	0	0.0	0	0.0	0	0.0

	Category	Total		RCT		Quasi-experiment		RCT protocol		MHealth product description		Cross-sectional study		Qualitative study		Case report	
		**N**	**%**	**N**	**%**	**N**	**%**	**N**	**%**	**N**	**%**	**N**	**%**	**N**	**%**	**N**	**%**
	Electronic Health Record check	1	0.4	0	0.0	0	0.0	0	0.0	1	100.0	0	0.0	0	0.0	0	0.0
	**Function for health service providers**																
	Data collection and management	37	15.1	4	10.8	8	21.6	0	0.0	14	37.8	9	24.3	2	5.4	0	0.0
	Decision support and guideline	18	7.3	0	0.0	8	44.4	0	0.0	8	44.4	2	11.1	0	0.0	0	0.0
	On-the-job training for health professionals	9	3.7	0	0.0	4	44.4	0	0.0	3	33.3	1	11.1	1	11.1	0	0.0
	Supervision and technical support	2	0.8	0	0.0	0	0.0	0	0.0	2	100.0	0	0.0	0	0.0	0	0.0
	Function for both																
	Counselling	38	15.5	4	10.5	6	15.8	2	5.3	21	55.3	5	13.2	0	0.0	0	0.0
**Disease**																	
	Infectious diseases	28	11.4	6	21.4	11	39.3	3	10.7	4	14.3	3	10.7	1	3.6	0	0.0
	Chronic diseases	43	17.6	6	14.0	11	25.6	6	14.0	17	39.5	2	4.7	1	2.3	0	0.0
	Mental and behavioural disorders	11	4.5	2	18.2	2	18.2	2	18.2	3	27.3	1	9.1	0	0.0	1	9.1
	Essential RMNCH issues	163	66.5	37	22.7	37	22.7	8	4.9	63	38.7	15	9.2	3	1.8	0	0.0
	Total	245	100.0	51	20.8	61	24.9	19	7.8	87	35.5	21	8.6	5	2.0	1	0.4
**Stages**																	
	Pre-pregnancy	26	10.6	10	38.5	2	7.7	2	7.7	12	46.2	0	0.0	0	0.0	0	0.0
	Pregnancy	88	35.9	14	15.9	24	27.3	4	4.5	36	40.9	8	9.1	2	2.3	0	0.0
	Delivery	9	3.7	1	11.1	1	11.1	0	0.0	7	77.8	0	0.0	0	0.0	0	0.0
	Postpartum	19	7.8	3	15.8	4	21.1	3	15.8	9	47.4	0	0.0	0	0.0	0	0.0

Category	Total		RCT		Quasi-experiment		RCT protocol		MHealth product description		Cross-sectional study		Qualitative study		Case report	
	**N**	**%**	**N**	**%**	**N**	**%**	**N**	**%**	**N**	**%**	**N**	**%**	**N**	**%**	**N**	**%**
Child care	139	56.7	24	17.3	32	23.0	11	7.9	53	38.1	14	10.1	4	2.9	1	0.7
